# Lignin-derived carbon nanosheets boost electrochemical reductive amination of pyruvate to alanine

**DOI:** 10.1016/j.isci.2023.107776

**Published:** 2023-08-29

**Authors:** Shunhan Jia, Xingxing Tan, Limin Wu, Ziwei Zhao, Xinning Song, Jiaqi Feng, Libing Zhang, Xiaodong Ma, Zhanrong Zhang, Xiaofu Sun, Buxing Han

**Affiliations:** 1 Beijing National Laboratory for Molecular Sciences, Key Laboratory of Colloid and Interface and Thermodynamics, Center for Carbon Neutral Chemistry, Institute of Chemistry, Chinese Academy of Sciences, Beijing 100190, China; 2 School of Chemical Sciences, University of Chinese Academy of Sciences, Beijing 100049, China; 3 Shanghai Key Laboratory of Green Chemistry and Chemical Processes, School of Chemistry and Molecular Engineering, East China Normal University, Shanghai 200062, China

**Keywords:** Electrochemical materials science, Nanoelectrochemistry, Applied chemistry

## Abstract

Efficient and sustainable amino acid synthesis is essential for industrial applications. Electrocatalytic reductive amination has emerged as a promising method, but challenges such as undesired side reactions and low efficiency persist. Herein, we demonstrated a lignin-derived catalyst for alanine synthesis. Carbon nanosheets (CNSs) were synthesized from lignin via a template-assisted method and doped with nitrogen and sulfur to boost reductive amination and suppress side reactions. The resulting N,S-co-doped carbon nanosheets (NS-CNSs) exhibited outstanding electrochemical performance. It achieved a maximum alanine Faradaic efficiency of 79.5%, and a yield exceeding 1,199 μmol h^−1^ cm^−2^ on NS-CNS, with a selectivity above 99.9%. NS-CNS showed excellent durability during long-term electrolysis. Kinetic studies including control experiments and theoretical calculations provided further insights into the reaction pathway. Moreover, NS-CNS catalysts demonstrated potential in upgrading real-world polylactic acid plastic waste, yielding value-added alanine with a selectivity over 75%.

## Highlights

• Lignin NS-CNS catalyst enables 79.5% efficient electrocatalytic alanine synthesis
• N,S-co-doping boosts amination and suppresses side reactions in alanine synthesis
• Durable NS-CNS ensures lasting amino acid production performance
• NS-CNS upgrades polylactic acid waste to value-added alanine

## Graphical abstract

**Figure:**
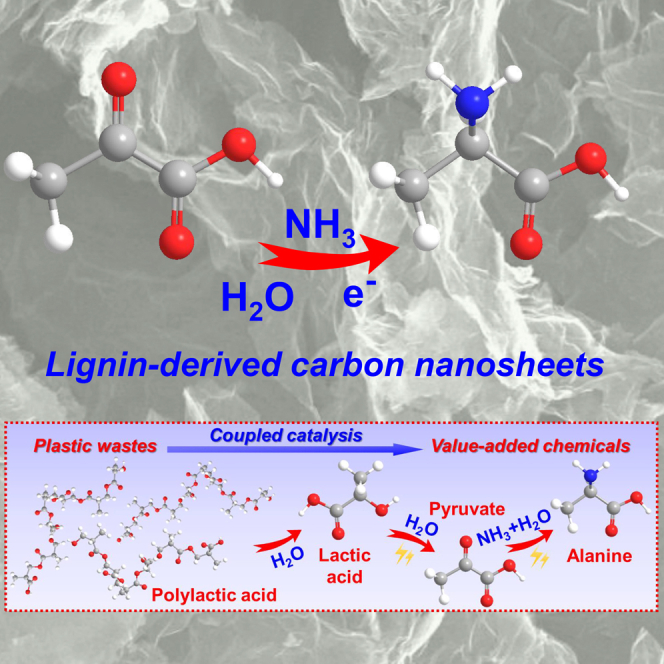

## Introduction

Amino acids play a crucial role in a variety of application fields, including pharmaceuticals, human and animal nutrition, and biomedical research.^1^^,^^2^^,^^3^^,^^4^ Consequently, the development of efficient and sustainable techniques for amino acid synthesis is of importance. Several approaches have been proposed, including biochemical, thermal chemical, and photo/electrochemical methods.^5^^,^^6^^,^^7^^,^^8^^,^^9^^,^^10^^,^^11^^,^^12^^,^^13^ Electrocatalytic reductive amination has shown remarkable potential in synthesizing amino acids from keto acids and nitrogen sources, such as ammonia (NH_3_), via C-N coupling.^14^^,^^15^^,^^16^^,^^17^ This process involves the formation of imine intermediates in the bulk electrolyte through the C-N coupling between carbonyl molecules and NH_3_, which could be reduced to amine products at the interface of electrocatalysts and electrolyte.^18^^,^^19^ Electrocatalytic reductive amination has many advantages, such as mild reaction conditions, renewable carbon sources, green electrons as reductants, and water as proton source.^20^^,^^21^^,^^22^^,^^23^^,^^24^ However, the direct reduction of carbonyl compounds and the hydrogen evolution reaction (HER) are undesired competing side reactions, which could reduce the Faradaic efficiency (FE) and yield rates of amino acids. Therefore, there is a strong need for the design of novel and effective catalysts to selectively reduce imine intermediates and inhibit HER in the synthesis of amino acids through electrocatalytic reductive amination.^25^^,^^26^^,^^27^

Carbon materials are garnering growing attention in the field of electrocatalysis and emerging as promising alternatives to conventional metal-based catalysts.^28^^,^^29^^,^^30^^,^^31^ This is due to their excellent properties, such as high surface area, good conductivity, excellent stability, low cost, and eco-friendliness. The advantages of carbon materials with 2D morphology include an extensive surface area with abundant active sites, facilitating efficient catalytic reactions and higher reaction rates. The nanosheets' exceptional electrical conductivity reduces charge transport distance, leading to enhanced electrocatalytic activity. Additionally, their mechanical strength ensures structural stability, while chemical stability guarantees long-term functionality even under harsh conditions. The 2D structure further facilitates mass transport, allowing easy diffusion of reactants to the catalyst surface. These collective benefits position carbon nanosheets (CNSs) as a reliable, durable, and efficient option for various electrocatalytic applications. Moreover, these materials can be customized using a range of techniques, such as heteroatomic doping (e.g., N, S, P, Se, etc.), which allows for the adjustment of physicochemical properties, facilitating the absorption of specific intermediates on their surface.^32^^,^^33^^,^^34^ This customization process creates active sites for desired electrochemical reactions and impedes side reactions. For instance, previous study highlighted the enhanced activity, selectivity, and stability of heteroatom-doped carbon-based catalysis for CO_2_ reduction, water electrolysis, electrocatalytic ammonia synthesis, and other important reactions.^35^^,^^36^^,^^37^^,^^38^^,^^39^ Additionally, carbon materials can be derived from various sustainable carbon sources, such as CO_2_, biomass, and waste plastics.^40^^,^^41^^,^^42^ For example, lignin, a discarded waste product of the pulp and paper industry, has high carbon content, numerous functional groups, and a unique structure, making it an ideal carbon source for the synthesis of carbon-based catalysts.^43^^,^^44^^,^^45^ Therefore, the carbon materials derived from lignin offer a promising avenue for achieving carbon-neutral and high-performance electrocatalysis, which has significant implications for the reductive amination synthesis of amino acids.

Herein, we developed a catalyst derived from lignin, which proves to be highly effective in the synthesis of alanine (Ala). By utilizing lignin as a sustainable carbon source, we synthesized CNSs through a template-assisted method. The introduction of nitrogen and sulfur dopants (N,S-co-doping) was found to be a promising strategy for enhancing the reductive amination of pyruvate to Ala, while simultaneously mitigating HER side reactions. The catalytic system based on N,S-co-doped carbon nanosheets (NS-CNSs) demonstrated remarkable performance in the electrochemical synthesis of Ala. Under a potential of −0.3 V versus reversible hydrogen electrode (vs. RHE, all potentials are with reference to RHE), the FE of Ala reached 79.5%, while at −0.5 V, the yield of Ala could achieve as high as 1,199 μmol h^−1^ cm^−2^ on NS-CNS with a selectivity of >99.9%. Importantly, NS-CNS exhibited excellent durability during long-term electrolysis, ensuring its suitability for practical applications. Furthermore, control experiments and theoretical calculations were conducted to reveal the mechanism. Expanding on the potential applications of NS-CNS catalysts, we explored their utilization in a coupled strategy to upgrade real-world polylactic acid (PLA) plastic waste, yielding value-added Ala with a selectivity exceeding 75%.

## Results and discussion

**Figure 1:**
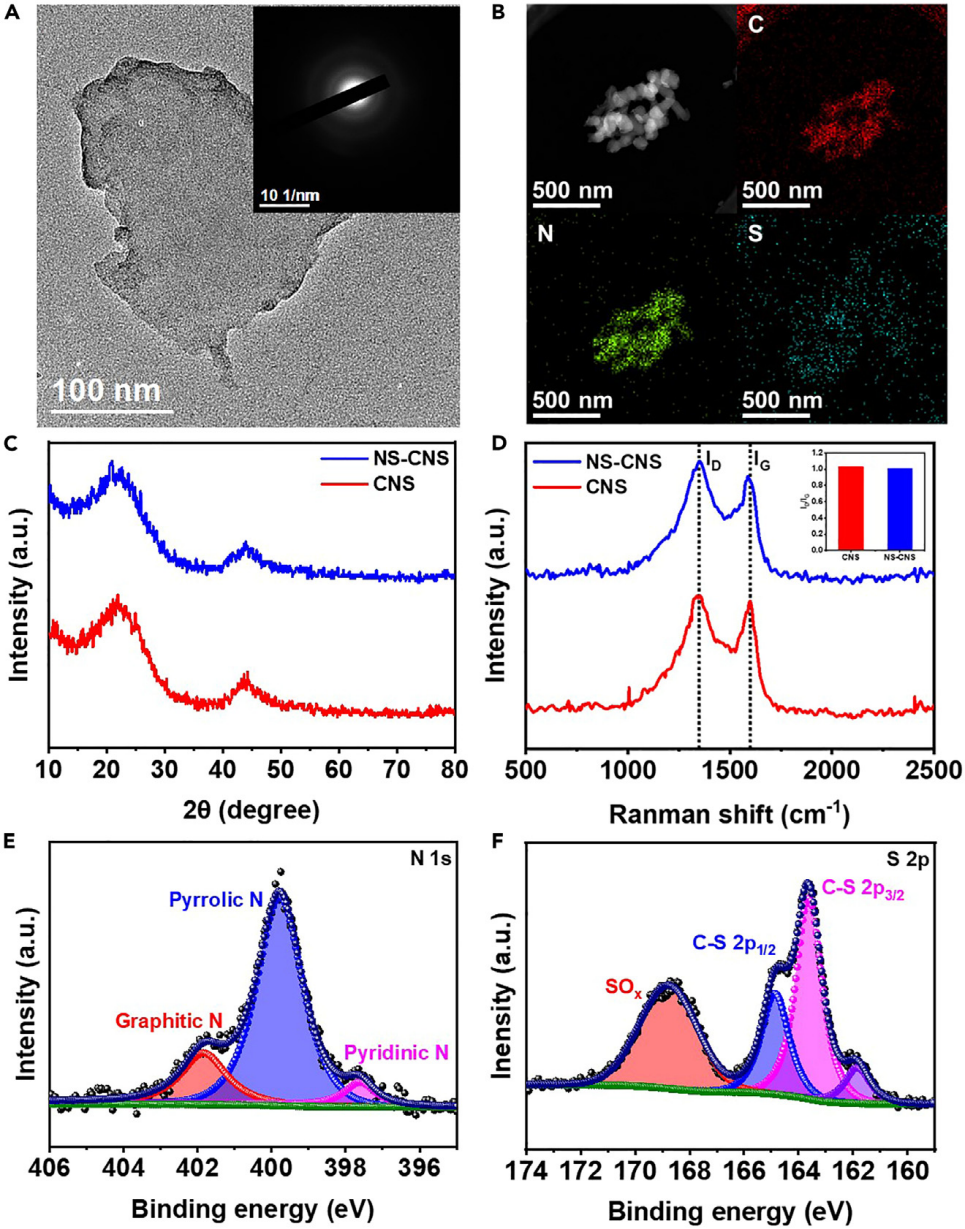
Carbon nanosheet catalysts (A and B) (A) HR-TEM image (inset is the corresponding SAED) and (B) EDX mapping images of NS-CNS catalyst. (C) XRD patterns of NS-CNS and CNS samples. (D) Raman spectra of NS-CNS and CNS. (E and F) XPS spectra of NS-CNS in the regions of (E) N 1s and (F) S 2p.

Cedar lignin underwent extraction through acid catalysis, resulting in a transformation from a light-yellow powder (Figure S1) to a reddish-brown powder (Figure S2). Mg_5_(CO_3_)_4_(OH)_2_·4H_2_O (as is shown in Figures S3–S5) was used as the template to synthesize biomass-based catalysts. The modified form of lignin served as the precursor for the catalyst. By subjecting a homogeneous mixture of a Mg_5_(CO_3_)_4_(OH)_2_·4H_2_O template and lignin to calcination at 900°C under Ar atmosphere for 1 h, CNSs were obtained following by removing templates through acid washing. Further, NS-CNSs were achieved through a combination of hydrothermal treatment and subsequent re-calcination, utilizing thiourea as the source of both N and S. Remarkably, the as-prepared CNS and NS-CNS, exhibited a sheet-like structure that mirrors the morphology of Mg_5_(CO_3_)_4_(OH)_2_·4H_2_O templatas preparede from the scanning electron microscopy (SEM) images (Figures S6 and S7). The transmission electron microscopy (TEM) images in Figure 1A provided evidence that NS-CNS consists of amorphous CNSs, while CNS possesses a similar morphology to NS-CNS (Figure S8). Furthermore, the high-angle annular bright field (HAABF) image, accompanied by corresponding element mapping, confirmed the homogeneous distribution of carbon (C), nitrogen (N), sulfur (S), and trace oxygen (O) within the NS-CNS samples (Figures 1B and S9), while only C and O were observed on CNS samples as shown in Figure S9. The 2D structure of NS-CNS could expose the catalytic sites to promote reductive amination.

X-ray diffraction (XRD) patterns in Figure 1C revealed two broad peaks around 21.6° and 43.4°, corresponding to the (002) and (100) planes of graphitic C. Raman spectroscopy investigations were performed to assess crystallinity, wherein distinct D and G bands were observed at approximately 1,345 cm^−1^ and 1,588 cm^−1^, respectively, in NS-CNS and CNS. These bands represent disordered sp^2^ hybridized carbon and ordered carbon structures, respectively. Notably, the intensity ratio of the D and G bands (I_D_/I_G_) serves as an indicator of the degree of disorder in the graphite structure. The degree of disorder in both NS-CNS (1.01) and CNS (1.03) was comparable, suggesting the unchanged crystallinity of the carbon catalysts after the introduction of N and S.

X-ray photoelectron spectroscopy (XPS) was employed to investigate the composition and chemical properties of the catalysts, as depicted in Figure S10. The N 1s spectra of NS-CNS exhibited three distinct components, namely pyridinic N (397.6 eV), pyrrolic N (399.8 eV), and graphitic N (401.8 eV), as illustrated in Figure 1E. Additionally, the S 2p spectra could be separated into three principal constituents (Figure 1F), including C-bonded S 2p_3/2_ (C-S 2p_3/2_, 163.6 eV), C-bonded sulfur 2p_1/2_ (C-S 2p_3/2_, 164.8 eV), and oxidized sulfur species (SO_x_ 168.8 eV). The NS-CNS samples contained 3.5 atom % of N and 2.5 atom % of S. Furthermore, CNS specimens were also subjected to XPS characterization (Figures S11 and S12), which revealed the absence of any detectable N and S species.

**Figure 2:**
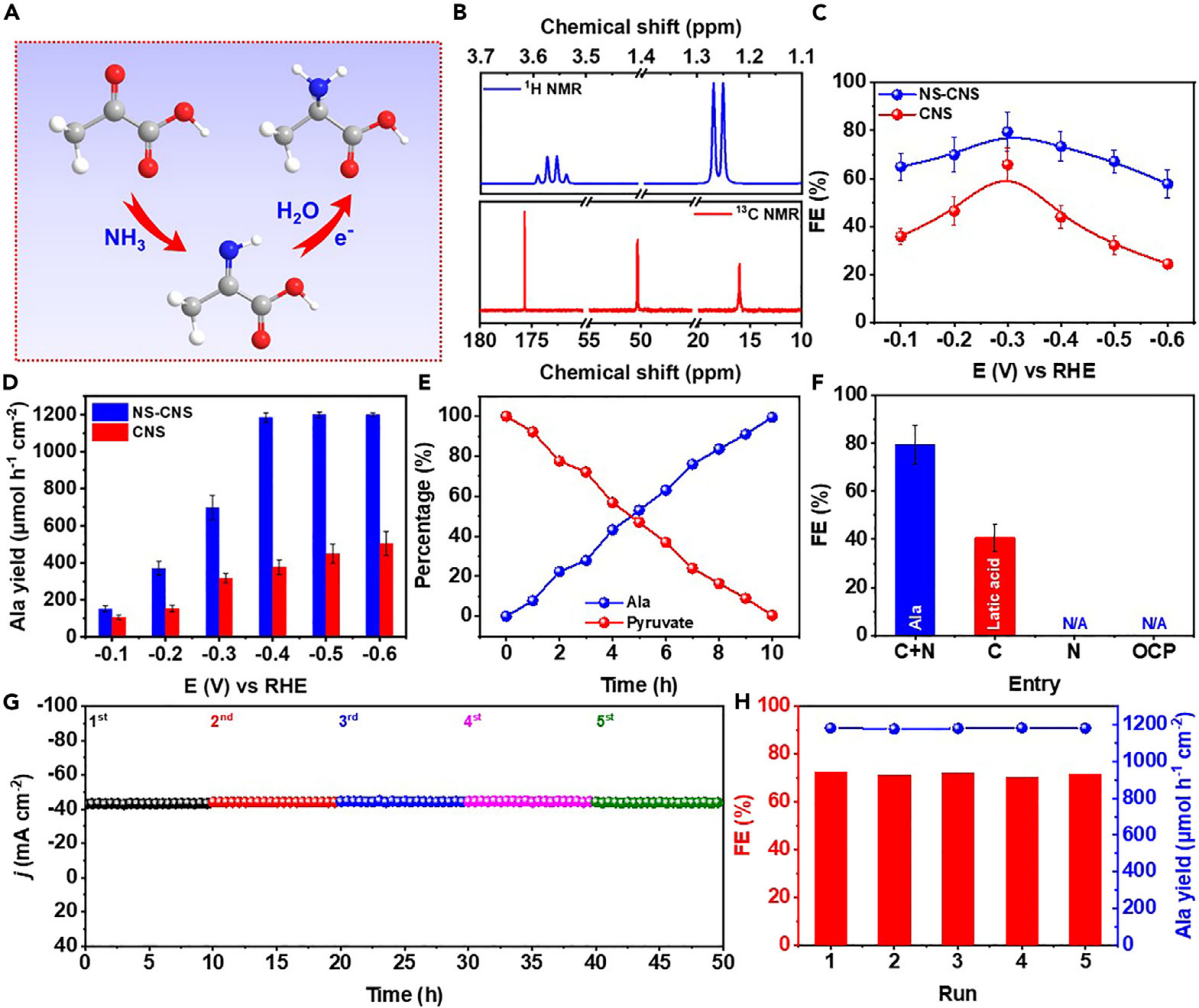
Electrochemical performance of reductive amination (A) Reaction pathway of electrocatalytic reductive amination. (B) ^1^H NMR and ^13^C NMR spectra of Ala products. (C) FE of Ala production on NS-CNS and CNS catalysts. (D) Yield rate of Ala on different catalysts. (E) Kinetic curve of time-dependance Ala yield on NS-CNS at −0.3 V. (F) Yields of Ala and other products using different electrolyte on NS-CNS catalyst at −0.3 V. C + N, the optimal reductive amination condition. C, Remove nitrogen source. N, Remove the carbon source. (G and H) (G) Chronoamperometry curves and (H) corresponding FE and yield rate of Ala for electrocatalytic reductive amoniation on NS-CNS at −0.3 V during consecutive recycling tests. Error bars are shown in the figure.

The electrochemical performance of NS-CNS and CNS was assessed in a 2 M NH₃/(NH₄)₂SO₄ buffer solution (pH = 10) containing 40 mM pyruvate, saturated with Ar gas. An H-type cell (Figure S13) was used for the experimental setup. All recorded potentials were referenced to the relative hydrogen electrode (RHE), and the error bars represent the standard deviation of three independent measurements. To construct the working electrode, a homogeneous ink of carbon-based catalysts was prepared and evenly loaded onto carbon paper at a loading of 1 mg cm⁻^2^. The electrocatalytic performance of reductive amination shown in Figure 2A, wherein a carbonyl group undergoes transformation into an amine through the intermediary of an imine, was initially investigated using linear scanning voltammetry (LSV) with a scan rate of 10 mV s⁻^1^, as depicted in Figure S14. The addition of 40 mM pyruvate resulted in an enhanced current density on both NS-CNS and CNS, suggesting the superior performance of NS-CNS over CNS in the context of the reductive amination of pyruvate to Ala. To explore the optimal efficiency of Ala production, chronoamperometry experiments were conducted by applying a range of potentials from −0.1 to −0.6 V. The stability of these chronoamperometry curves during the 10-h electrochemical tests was demonstrated in Figure S15. The corresponding ^1^H nuclear magnetic resonance (NMR) and^1^ ³C NMR spectra presented in Figure 2B confirm the formation of Ala molecules. Furthermore, the successful synthesis of Ala was also corroborated by determining the molecular weight of 88.1 Da using liquid chromatography-mass spectrometry (LC-MS). The FEs of Ala and yield were showcased in Figure 2C. Notably, NS-CNS exhibited the highest FE of 79.5% at −0.3 V, accompanied by an Ala yield of 698 μM h⁻^1^ cm⁻^2^. In addition, in Figure 2D, the potential of −0.4 V led to >99.9% conversion of pyruvate and >99.9% selectivity of Ala (based on the carbon source). At more negative potentials, the FE of Ala decreased due to increased hydrogen evolution, which competed with the reductive amination process. The FE of H₂ by-products could be observed in Figures S16 and S17, displaying a reverse trend to the FE of Ala. Moreover, NS-CNS demonstrated significantly higher FE and yield of Ala compared to undoped CNS, indicating that the electrochemical performance could be greatly enhanced through N and S co-doping. Furthermore, the reaction process was monitored, as depicted in Figure 2E. Within a span of 10 h, pyruvate was fully consumed, and the yield of Ala exhibited an opposite trend to that of pyruvate. A carbon-based catalyst derived from lignin, without undergoing extraction, was subjected to testing, revealing the prevalence of carbon nanoparticles with notably large particle diameters in the context of a graphene π-system; C atoms positioned proximate to N and S atoms acquire a positive charge owing to the electron-withdrawing nature of these heteroatoms. Consequently, such positively charged C centers in close proximity to heteroatoms exhibit a pronounced propensity to readily engage in the absorption of imine intermediates. As a result, NS-CNS could exhibit enhanced performance than CNS. The catalyst displayed an exceedingly low selectivity (<1%) toward the desired Ala product. This observation underscores the fundamental significance of lignin extraction in the production of highly efficient catalysts for reductive amination processes.

To eliminate the influence of other nitrogen sources that may generate Ala, such as nitrogenous catalysts and other impurities, control experiments were conducted in Figure 2F. No Ala production was detected in the cathode solution after electrolysis without a nitrogen source or carbon source, as well as at the open circuit potential (OCP), indicating that the nitrogen source of Ala products was NH_3_ (NH_4_^+^) in the electrolyte. Moreover, it was observed that the FE of Ala, under optimal conditions, surpassed that of lactic acid in the absence of a nitrogen source, implying an augmented reduction of amine intermediates in contrast to ketone intermediates on the NS-CNS catalyst. The limited adsorption of ketone species on the surface of NS-CNS was postulated as a plausible explanation for the absence of lactic acid by-products observed under the optimized conditions.

Furthermore, the NS-CNS cathode can be easily recycled and reused after washing with ethanol and water. The chronoamperometry curves at −0.4 V in Figure 2G remained stable throughout five reutilizations. Notably, as shown in Figure 2H, even in the fifth run, conducted under optimal reaction conditions, an FE of 71.7% and a yield of 1,181 μM h⁻^1^ cm⁻^2^ were achieved, demonstrating the remarkable stability and efficiency of the NS-CNS catalyst (Figure 2H). Based on LSV (Figure S18), XRD (Figure S19), SEM (Figure S20), and TEM (Figure S21) analysis of NS-CNS following 50 h of electrolysis, the crystalline phase of carbon remained largely unaltered, and the morphology exhibited good preservation. Furthermore, the XPS spectra (Figures S22–S24) illustrated that the surface valence state and contents of N and S atoms on the surface of NS-CNS remained nearly unchanged compared to the initial catalyst after the cycling test. These findings serve as evidence that NS-CNS demonstrates remarkable stability for electrocatalytic reductive amination.

**Figure 3:**
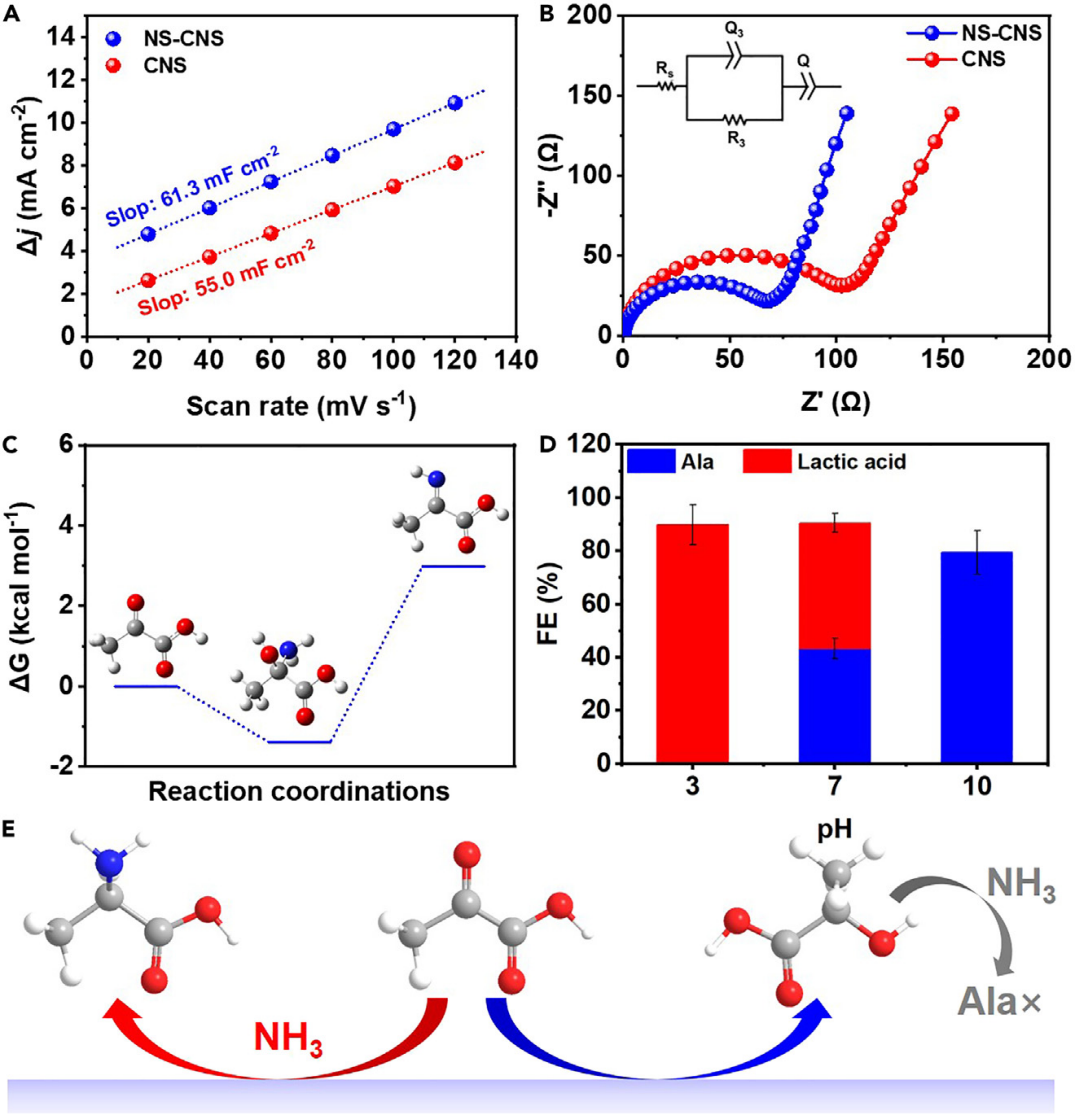
Kinetics study of reductive amination (A) Charge current density differences plotted against the scan rates. (B) Nyquist plots for different catalysts in reductive amination. (C) Energy diagram of the formation of imine intermediate. (D) FE of Ala and lactic acid under different pH. (E) Reaction pathway of reductive amination. Error bars are shown in the figure.

We further investigated the kinetics of reductive amination over NS-CNS. Figure 3A illustrates the determination of the electrochemically active surface area for both NS-CNS and CNS, calculated through the double-layer capacitance (C_dl_) based on the non-Faraday region cyclic voltammetry curves (as depicted in Figures S25 and S26). The calculated C_dl_ value for NS-CNS (61.3 mF cm^−2^) surpassed that of CNS (55.0 mF cm^−2^), providing additional evidence that N,S-co-doping contributed to the enhanced exposure of active sites for the electrochemical synthesis of Ala. The higher C_dl_ value of NS-CNS compared to CNS as an electrochemical catalyst for pyruvate reduction to Ala is attributed to various factors: increased surface area due to N and S dopants, improved charge transfer kinetics, and specific active sites favoring the reaction, potentially enhanced by a synergistic effect between the dopants. Electrochemical impedance spectroscopy (EIS) was employed to assess the charge transfer resistance (R_ct_) of the samples at the open circuit potential in an argon-saturated electrolyte. The Nyquist plots presented in Figure 3B demonstrate that NS-CNS exhibited the most favorable interfacial R_ct_ value, suggesting that electron transfer is more facile on NS-CNS compared to CNS. Typical carbon features with equivalent series resistance (ESR), equivalent distributed resistance (EDR), and capacitance were also shown in Figure 3B.^46^^,^^47^ The ESR primarily arises from the resistance of the electrolyte solution and electrical connections. Moving to the Nyquist plot’s high-frequency region, the observed arc is a consequence of limitations on ion transport through the carbon/Nafion matrix, termed as EDR. Following the high-frequency region, a straight line parallel to the y axis with a slight tilt toward the x axis becomes apparent in the mid-frequency and low-frequency regions. This straight line is attributed to the double-layer capacitance. In comparison, it is noteworthy that CNS exhibits a higher EDR value when contrasted with NS-CNS. Slight change of Nyquist plots was observed before and after electrolysis for NS-CNS (Figure S27), further confirming the stability of NS-CNS during long-term reductive amination. In the context of a graphene π-system, C atoms positioned proximate to nitrogen and S atoms acquire a positive charge owing to the electron-withdrawing nature of these heteroatoms.^48^^,^^49^^,^^50^ Consequently, such positively charged C centers in close proximity to heteroatoms exhibit a pronounced propensity to readily engage in the absorption of imine intermediates. As a result, NS-CNS could exhibit enhanced performance than CNS.

In addition, density functional theory (DFT) calculations were employed to investigate the kinetics of reductive amination. Figure 1A illustrates the formation of an imide intermediate resulting from the interaction between pyruvate and ammonia in the bulk of solution, subsequently undergoing electroreduction on the surface of NS-CNS. Notably, Figure 3C reveals that the corresponding imine intermediate, originating from pyruvate, experienced a minimal energy increase of merely 2.98 kcal mol^−1^. This energy barrier suggests that the formation of the imine intermediate could occur at room temperatures. Additionally, upon scrutinizing the reactant complex (Figure S28) with a prolonged C-N bond scan shown in Figure S29, it was observed that the energy consistently rises without attaining a stable product structure. Consequently, the direct attack of NH_3_ on the carbonyl carbon, facilitated by the coordination of base molecules, was deemed as the viable pathway. This finding aligns with experimental results, which demonstrate a low yield rate of the reductive amination reaction under acidic conditions (Figure 3D). The presence of NH_3_ (NH_4_^+^) in the solution not only serves as a nitrogen source for Ala generation but also acts as a buffer, providing an appropriate pH for reductive amination. This synergistic effect between the solution and the highly active NS-CNS catalyst contributed to the efficient production of Ala. In our pursuit to deepen our comprehension of the pathway involved in reducing aminopyruvate to Ala, we undertook a comparative experiment. Under standard conditions, we observed a remarkable absence of Ala products when lactic acid was utilized as the carbon source. Furthermore, when pyruvate, ammonia, and electrolytic conditions were removed, no Ala products were produced. These observations led us to investigate further, and our subsequent findings confirmed the central role of pyruvate-derived imine intermediates in the reduction amination process. Specifically, these imine intermediates were subjected to electroreduction on the surface of NS-CNS, resulting in the successful production of Ala. This discovery sheds light on the vital role played by NS-CNS in facilitating Ala synthesis through the electroreduction of pyruvate-derived imine intermediates, as shown in Figure 3E.

**Figure 4:**
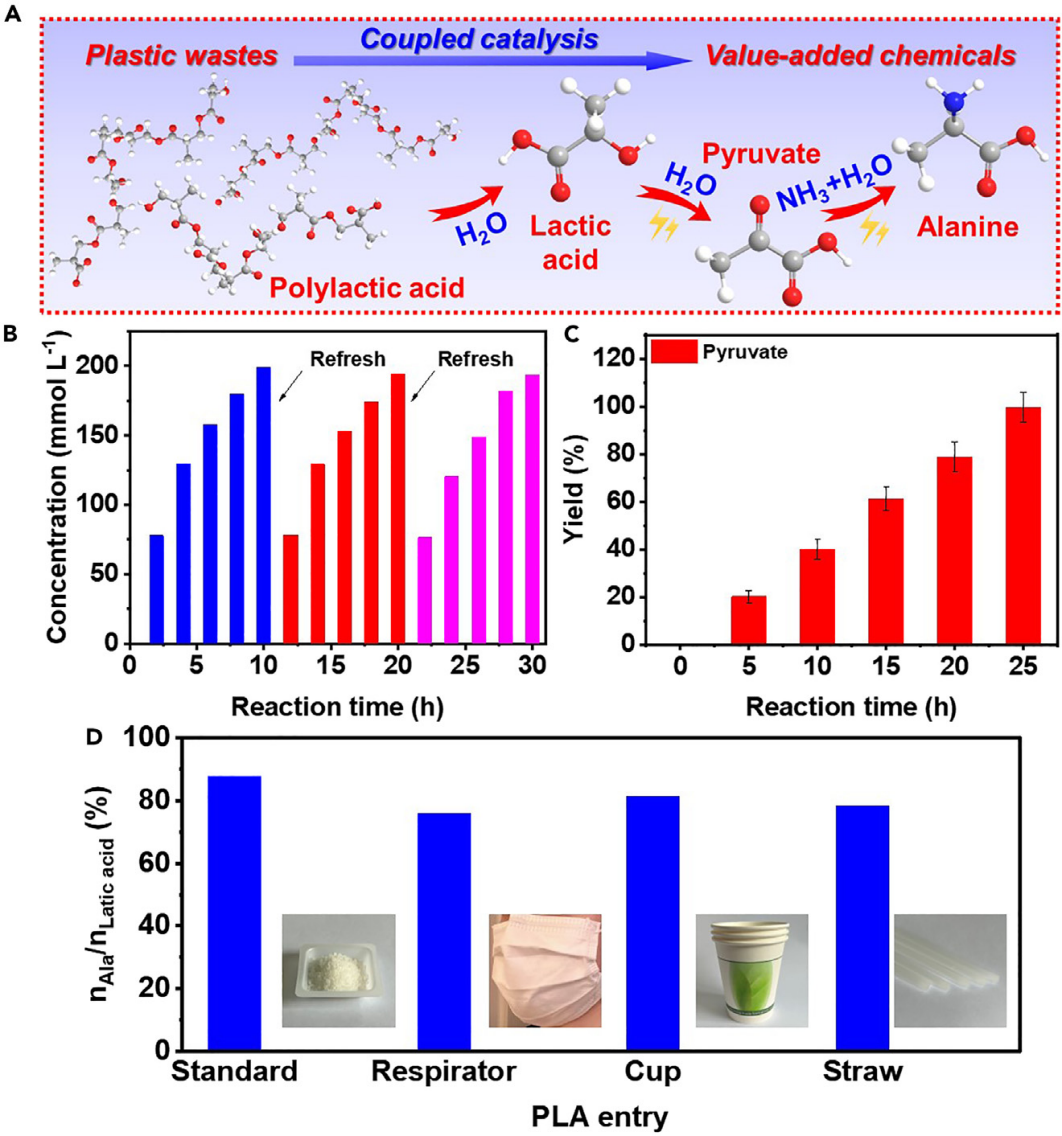
Coupled catalysis for PLA to Ala (A) Illustration of the reaction pathway of the upcycling of PLA to Ala. (B) Concentration of lactic acid produced from PLA hydrolysis for different reaction time. (C) Yield of pyruvate from the electrooxidation of lactic acid with water on IrO_2_/Ti electrode for different reaction time. (D) Production of Ala for different tandem catalysis pathway from different real PLA wastes. Error bars are shown in the figure.

In the light of the potential to obtain pyruvate from waste PLA plastics through straightforward hydrolysis and electrooxidation, we employed the NS-CNS catalyst to facilitate the conversion of pyruvate derived from PLA waste, thus establishing a tandem conversion chain from waste plastics to amino acids (Figure 4A). PLA has become the preferred choice for biodegradable plastics compared to traditional petroleum-based alternatives, owing to its propensity to break down into carbon dioxide and water in the environment.^51^^,^^52^^,^^53^ However, the natural degradation of PLA is a prolonged process, accompanied by CO_2_ emissions^51^ Consequently, there is an urgent need to develop an innovative conversion approach to upgrade plastic waste into high value-added products, particularly those exhibiting superior chemical selectivity.

In our initial investigation, we conducted a comprehensive study employing standard PLA samples. The hydrolysis of PLA (Figure S30) was performed in a hermetically sealed Teflon reactor under ambient atmospheric conditions, maintaining a temperature of 80°C. We observed a gradual increase in the concentration of lactic acid in the solution over time, accompanied by a deceleration in the rate of increase (Figure 4B). This could be attributed to the presence of lactic acid molecules in the solution, which exert an inhibitory effect on further hydrolysis reactions. However, upon refreshing the reaction solution (Figure S31), the hydrolysis rate of PLA returns to its initial state. Remarkably, after a hydrolysis period of 10 h, a concentrated lactic acid solution with a concentration of 200 mmol L^−1^ was generated, rendering it suitable for subsequent conversion reactions following appropriate dilution. The lactic acid solution obtained from hydrolysis was subjected to electrooxidation for the conversion into pyruvate (Figure S32), utilizing an IrO_2_/Ti electrode, a commonly employed catalyst in electrochemical oxidation.^52^ LSV analysis presented in Figure S33 confirmed the occurrence of the oxidation reaction on IrO_2_ catalyst, as evidenced by the decrease in initial potential and the subsequent increase in current upon the addition of lactic acid to the electrolyte. To produce pyruvate, we selected a potential of +0.6 V vs. RHE after optimizing the yield of pyruvate under different applied potentials (Figure S33). Over the course of electrolysis, the concentration of pyruvate in the electrolyte demonstrated a linear increase with respect to the electrolysis time. Remarkably, after a reaction period of 25 h, we achieved a remarkable selectivity of over 99.9% toward pyruvate (Figure 4C). Following pH adjustment of the electrolyte and the introduction of a nitrogen source, the resultant electrolyte was directly employed in an NS-CNS- catalyzed reduction amination reaction to produce Ala. After the investigation concerning CN-CNS-catalyzed pyruvate reductive amination leading to Ala production, we have achieved a remarkable selectivity of 88% for the conversion of lactic acid monomers to Ala in PLA standard samples. Motivated by these promising results, we further explored the conversion of PLA waste derived from real-world sources into Ala, as is shown in Figure 4D. Specifically, PLA-based respirators, water cups, and straws were utilized in this study, yielding selectivity from lactic acid monomers to Ala products of 76%, 81%, and 78%, respectively. These findings provide conclusive evidence that the sequential pathway we have established exhibits superior selectivity for converting real-word PLA wastes plastics into value-added Ala products.

### Conclusion

In summary, we developed a lignin-derived NS-CNS catalyst for efficient and sustainable synthesis of Ala through electrocatalytic reductive amination. The incorporation of N,S-co-doping proved to boost the reductive amination of pyruvate to Ala, while simultaneously suppressing undesired HER. NS-CNS exhibited remarkable electrochemical performance for Ala synthesis. At a potential of −0.3 V, we achieved a high FE of 79.5% for Ala production, and at −0.5 V, the yield of Ala reached an impressive rate exceeding 1,199 μmol h^−1^ cm^−2^ on NS-CNS, with a selectivity exceeding 99.9%. Moreover, NS-CNS exhibited excellent durability during long-term electrolysis, ensuring its potential applicability. We conducted comprehensive kinetic studies, including control experiments and theoretical calculations, to gain insights into the reaction pathway of reductive amination. Furthermore, we explored the potential application of NS-CNS catalysts in the upgrade of real-world PLA plastic waste, resulting in the production of value-added Ala with a selectivity exceeding 75%. Our work demonstrated the significant potential of lignin-derived NS-CNS in achieving carbon-neutral and high-performance electrocatalysis for amino acid synthesis. These findings contribute to the development of efficient and sustainable techniques for amino acid production and utilization of sustainable carbon/nitrogen sources.

## STAR★Methods

### Key resources table

**Table:** 

REAGENT or RESOURCE	SOURCE	IDENTIFIER
**Chemicals, peptides, and recombinant proteins**		

1,4-dioxane	Sigma-aldrich	CAS 123-91-1
HCl	HCl	CAS 7647-01-0
acetone	Concord	CAS 67-64-1
diethyl ether	Concord	CAS 60-29-7
MgCl_2_·6H_2_O	Sigma-aldrich	CAS 7791-18-6
urea	Sigma-aldrich	CAS 57-13-6
CTAB	Sigma-aldrich	CAS 57-09-0
KOH	Sigma-aldrich	CAS 1310-58-3
NaOH	Sigma-aldrich	CAS 1310-73-2
thiourea	Sigma-aldrich	CAS 62-56-6
isopropanol	Concord	CAS 67-63-0
(NH_4_)_2_SO_4_	Sigma-aldrich	CAS 7783-20-2
deuterium oxide	Sigma-aldrich	CAS 7789-20-0
DSS	TCI	CAS 2039-96-5
lactic acid	Sigma-aldrich	CAS 50-21-5
pyruvate	Sigma-aldrich	CAS 127-17-3

**Software and algorithms**		

Origin 2022b	Origin Lab	https://www.originlab.com/
MestReNova	mestrelab	https://mestrelab.com/
Avantage	Thermo Fisher Scientific	https://www.thermofisher.cn/
Gaussian 09	Gaussian, Inc.	https://Gaussian.com/

### Resource availability

#### Lead contact

Further information and requests for resources and reagents should be directed to and will be fulfilled by the lead contact, Prof. Buxing Han (hanbx@iccas.ac.cn).

#### Materials availability

This work did not generate new unique reagents.

#### Data and code availability

• All data reported in this paper will be shared by the lead contact upon request.
• This paper does not report original code.
• Any additional information required to reanalyze the data reported in this paper is available from the lead contact upon request.

### Experimental model and subject details

This work did not involove experimental model and subject.

### Method details

#### Extraction of cedar lignin

Lignin was extracted from cedar wood using a previously reported method. Briefly, cedar wood powders were dried under vacuum at 80°C for 24 h.^12^^,^^54^ Subsequently, 200 g of dried cedar wood powder, 1.5 L of 1,4-dioxane, and 2 M HCl (160 mL) were added to a 3 L three-necked flask. The mixture was heated to 110°C and held for 60 min. After cooling to room temperature, the mixture was filtered and washed with 1,4-dioxane. The resulting brownish-red liquid was concentrated to approximately 300 mL of gummy liquid, which was further treated with an acetone/water mixture (v/v = 9:1, ∼250 mL). The resulting mixture was then slowly added to rapidly stirring water (2.5 L). The formed precipitate was separated and dried under vacuum at 60°C for 24 h. The obtained material was re-dissolved in an adequate amount of acetone/methanol (9:1) mixture and precipitated by slowly pouring into rapidly stirring diethyl ether (2 L). The resulting red lignin precipitate was further dried under vacuum at 60°C for 72 h before use as a substrate for catalyst synthesis.

#### Synthesis of Mg_5_(OH)_2_CO_3_ template

The Mg_5_(CO_3_)_4_(OH)_2_·4H_2_O microsphere template for the synthesis of lignin-based catalysts were synthesized with minor modifications to a previously described method.^55^^,^^56^ Specifically, 15.0 g of MgCl_2_·6H_2_O, 20.0 g of urea, and 0.3 g of CTAB were dissolved in 250 mL of deionized H_2_O. The resulting solution was refluxed at 100°C for 12 h, followed by a 12 h standing period at 95°C. The resulting product was collected via centrifugation, washed with deionized H_2_O, and dried under vacuum at 60°C overnight.

#### Synthesis of catalysts

1.2 g of red lignin was dissolved in 50 mL of a mixture solution of NaOH (0.6 g) and KOH (0.6 g). The solution was stirred continuously for 6 h and dried in an oven at 80°C. 12 g of Mg_5_(CO_3_)_4_(OH)_2_·4H_2_O template was added to the dried solid product and stirred continuously. The resulting mixture was then heated in a tube furnace at 900°C for 1 h under Ar at the heating rate of 5°C min^−1^. The carbonized product was repeatedly washed with a 1 M HCl and dried to obtain the lignin-derived carbon nanosheets, CNS. 200 mg of CNS and 200 mg of thiourea were dispersed in 50 mL of distilled H_2_O. The solution was placed in an autoclave and reacted at 180°C for 12 h. The heated mixture was then filtered, and the precursor powder was obtained by drying overnight at 60°C in a blast oven under vacuum. The precursor powder was then carbonized at 800 under vacuum for 2 h under N_2_. Finally, the obtained material was labeled NS-CNS.

#### Material characterization

The microstructures of the catalystswere analyzed using scanning electron microscopy (SEM, HITACHI S-4800) and transmission electron microscopy (TEM, JEOL JEM-2100F) equipped with EDS. X-ray photoelectron spectroscopy (XPS) was carried out on the Thermo Scientific ESCALab 250Xi using a 200W Al-Kα radiation. The base pressure in the analysis chamber was maintained at about 3 × 10-10 mbar to ensure accurate results. The hydrocarbon C1s line at 284.8 eV was utilized for energy referencing. X-ray diffraction (XRD) analysis was performed on the samples using a Rigaku D/max-2500 X-ray diffractometer with Cu-Kα radiation (y = 0.15406 nm) at a scan speed of 5^o^ min^−1^. The Raman spectra of the samples were obtained on an FT Bruker RFS 106/S spectrometer equipped with a 514 nm laser in the region from 4000 to 100 cm^−1^ with a resolution of 2 cm^−1^, in a flame-sealed capillary at room temperature. The N_2_ adsorption/desorption isotherms were determined using a Micromeritics ASAP 2020 sorptometer operated at 77 K, and BET surface areas and pore volumes were obtained.

#### Electrochemical study of reductive amination

To fabricate the electrode, a catalyst ink was prepared by combining 1 mg of the catalyst with 200 μL of isopropanol and 10 μL of a 5 wt % Nafion dispersion. The mixture was sonicated for 30 min, and the resulting ink was slowly dispensed onto a carbon paper substrate using a micropipette to achieve a catalyst loading of approximately 1 mg cm^−2^. Electrochemical studies were conducted using a CHI660E electrochemical workstation in an H-type cell comprising a cathodic chamber, an anodic chamber, and a graphite rod counter electrode placed in the anodic chamber. The working electrode was placed in the cathodic chamber, and the two chambers were separated by an Nafion 117 membrane. Electrolyte solutions containing NH_3_/(NH_4_)_2_SO_4_ buffer (pH = 10) were utilized for the electrosynthesis of amino acids, with organic substrates added to the cathodic solution before the reaction. To eliminate atmospheric air, the cathodic electrolyte solution was purged with purified Ar gas for 30 min prior to each experiment, and Ar gas was continuously bubbled into the cathodic electrolyte solution during the measurements.

A Bruker Ascend 400 HD (400 MHz) instrument was utilized to perform ^1^H NMR spectroscopy on the collected reaction solution from the cathodic compartment. This analysis was conducted at room temperature. In each analysis, 500 μL of the electrolyte was mixed with 500 μL of deuterium oxide which contained 4,4-dimethyl-4-silapentane-1-sulfonic acid (DSS) used as an internal standard. The FE for each compound was calculated utilizing the following equation:

FE = (n × C × V × F)/Q × 100%where n was the number of electrons needed for the products to form, C was the molar concentration of the products, V was the volume of the electrolyte, F was the Faraday constant, and Q represented the total charge passed during the electrochemical test.

#### DFT calculations

All calculations were conducted using the Gaussian16 package. The M06-2X hybrid functional was employed for all computations. Geometry optimization was carried out utilizing the 6-31G(d,p) basis set.^57^ Analytical frequency calculations were performed at the same level of theory as the geometry optimization to determine the nature of each stationary point, distinguishing between minima (no imaginary frequencies) and transition states (only one imaginary frequency). Additionally, Gibbs free energy corrections at a temperature of 298.15 K were obtained. The final energies for the fully optimized structures were computed using the larger 6-311+G(d,p) basis set.

#### Hydrolysis of PLA samples

First, the PLA plastic waste was cut into small pieces mixed with water. The mixture was placed in a into a Teflon-lined stainless-steel reactor and stirred using a magnetic stirrer at a moderate speed. The mixture was heated to a temperature of 120°C and maintained at this temperature for 12 h. The yield of lactic acid was calculated using 1H NMR with DSS as the internal standard.

#### Electrocatalytic oxidation of lactic acid to pyruvate

The IrO_2_/Ti electrode was created using a method previously described with some modifications.^58^ The process began by etching the Ti mesh in boiling 5 M HCl for 1 h. Next, the Ti mesh was dip-coated in a solution composed of 2 mL HCl, 20 mL isopropanol, and 50 mg of IrO_2_. The resulting catalyst was then dried in a preheated oven at 100°C for 10 min and calcined in air at 500°C for an additional 10 min. To achieve an IrO_2_ loading of 1 mg cm^−2^, the entire process was repeated ten times. The electrolyte used for the experiment was a lactic acid aqueous solution (0.2 mol L^−1^) with 1 mol L^−1^ KOH serving as the supporting electrolyte. Additionally, 1 mol L^−1^ KOH solution was used as the cathode electrolyte with a Pt film electrode serving as the counter electrode. The yield of pyruvate was calculated using ^1^H NMR with the methods similar as described above.

### Quantification and statistical analysis

This work did not involove quantification and statistical analysis.
