# Supplementary material for: Lignin-derived carbon nanosheets boost electrochemical reductive amination of pyruvate to alanine
Source: iScience. 2023 Aug 29;26(10):107776. doi: 10.1016/j.isci.2023.107776 (PMC10502407; doi:10.1016/j.isci.2023.107776)
Supplement: Document S1. Figures S1–S33 [file mmc1.pdf]

iScience, Volume *26*

Supplemental information

Lignin-derived carbon nanosheets boost electrochemical reductive amination of pyruvate to alanine

Shunhan Jia, Xingxing Tan, Limin Wu, Ziwei Zhao, Xinning Song, Jiaqi Feng, Libing Zhang, Xiaodong Ma, Zhanrong Zhang, Xiaofu Sun, and Buxing Han

Lignin-derived carbon nanosheets boost electrochemical reductive amination of pyruvate to alanine

Shunhan Jia [1,2], Xingxing Tan [1], Limin Wu [1,2], Ziwei Zhao [1,2], Xinning Song [1,2], Jiaqi Feng [1], Libing Zhang [1,2], Xiaodong Ma [1], Zhanrong Zhang [1,2], Xiaofu Sun [1,2,], Buxing Han [1,2,3,4]**

[1] Beijing National Laboratory for Molecular Sciences, Key Laboratory of Colloid and Interface and Thermodynamics, Center for Carbon Neutral Chemistry, Institute of Chemistry, Chinese Academy of Sciences, Beijing 100190, China

[2] School of Chemical Sciences, University of Chinese Academy of Sciences, Beijing 100049, China

[3] Shanghai Key Laboratory of Green Chemistry and Chemical Processes, School of Chemistry and Molecular Engineering, East China Normal University, Shanghai 200062, China

[4] Lead contact. hanbx@iccas.ac.cn (B.H.)

* Corresponding author. Email: sunxiaofu@iccas.ac.cn (X.S.); hanbx@iccas.ac.cn (B.H.)

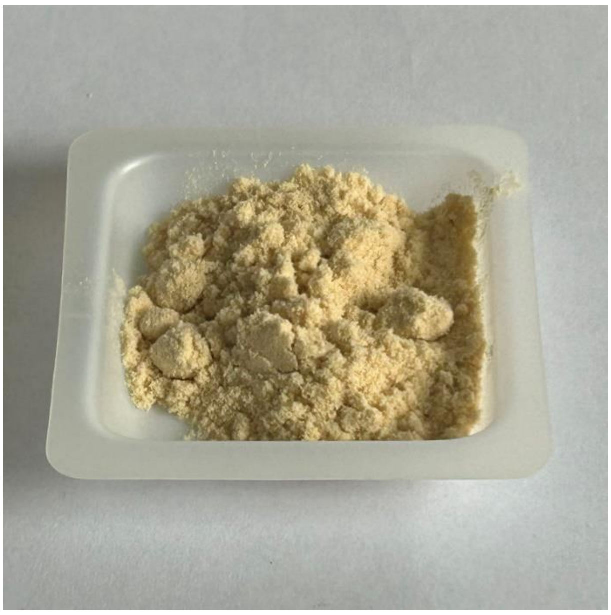

Figure S1. Optical photograph of initial Cedar lignin, related to Figure 1.

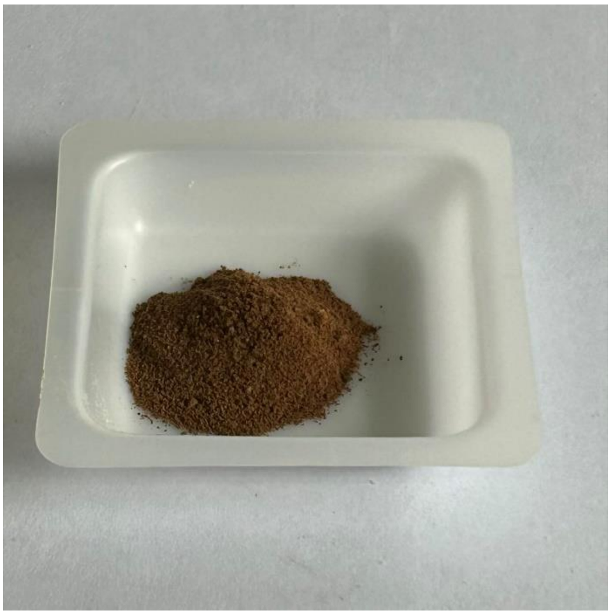

Figure S2. Optical photograph of extracted lignin powder, related to Figure 1.

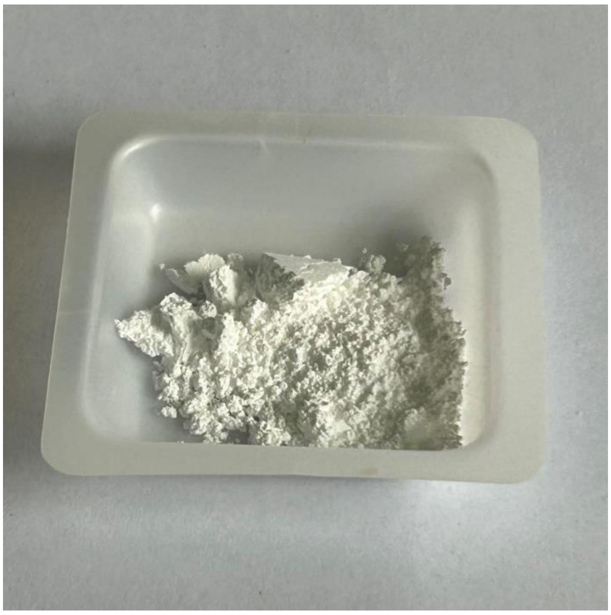

Figure S3. Optical photograph of the powder of $Mg_5(CO_3)_4(OH)_2 \cdot 4H_2O$ template, related to Figure 1.

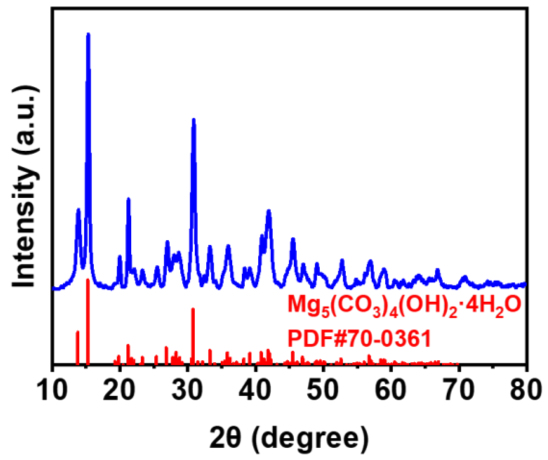

Figure S4. XRD pattern of $Mg_5(CO_3)_4(OH)_2 \cdot 4H_2O$ template, related to Figure 1.

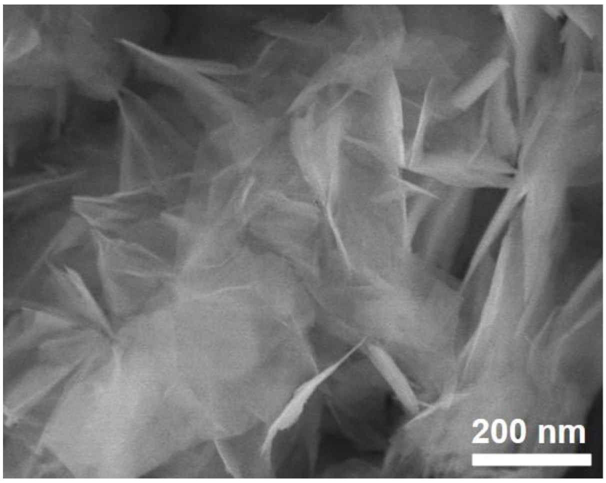

Figure S5. SEM image of the $Mg_5(CO_3)_4(OH)_2{\cdot}4H_2O$ template, related to Figure 1.

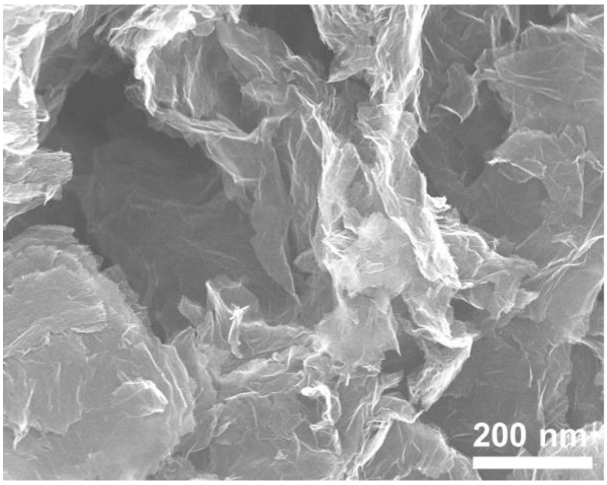

Figure S6. SEM image of CNS derived from lignin, related to Figure 1.

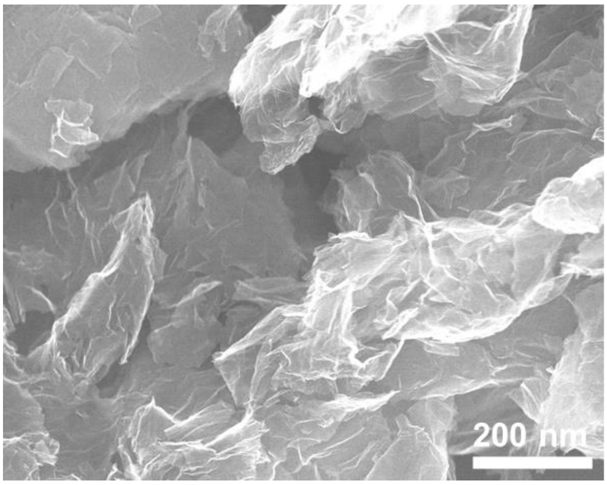

Figure S7. SEM image of NS-CNS samples, related to Figure 1.

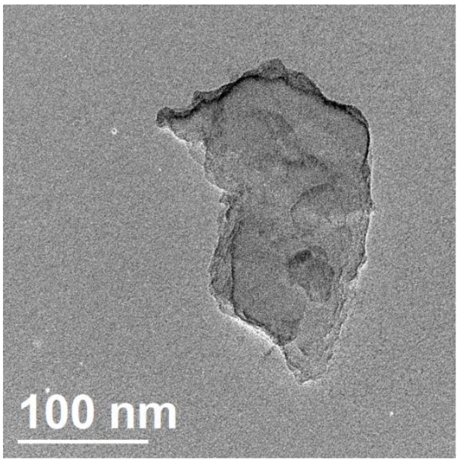

Figure S8. TEM image of CNS, related to Figure 1.

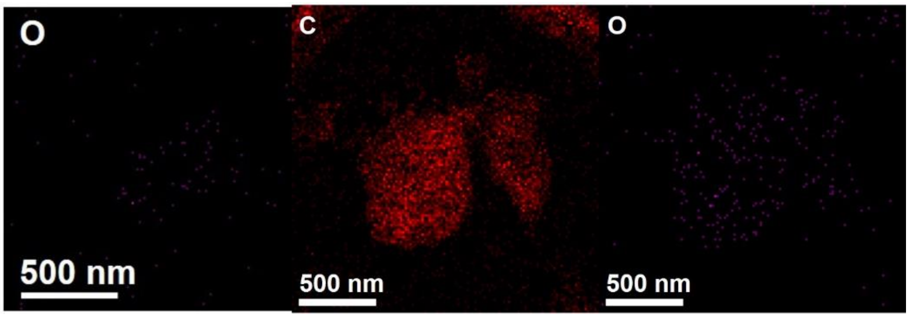

Figure S9. EDX mapping of O element of NS-CNS, and C and O element of CNS, related to Figure 1.

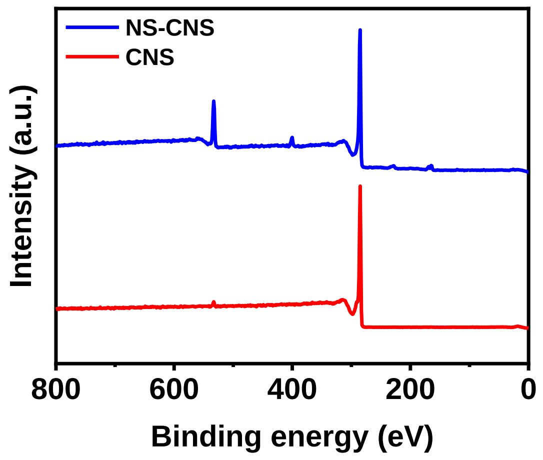

Figure S10. The overview XPS spectra of NS-CNS and CNS samples, related to Figure 1.

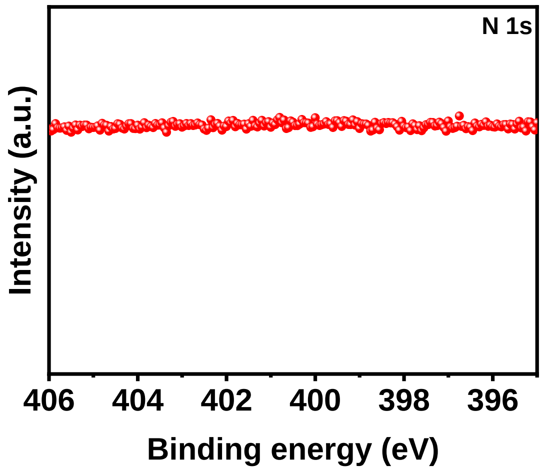

Figure S11. XPS spectra of CNS in the regions of N 1s, related to Figure 1.

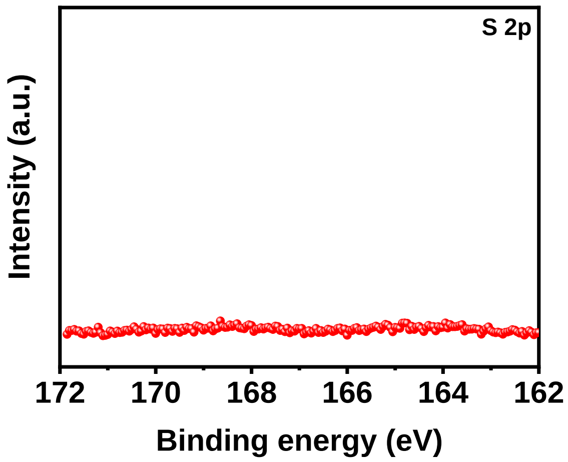

Figure S12. XPS spectra of CNS in the regions of S 2p, related to Figure 1.

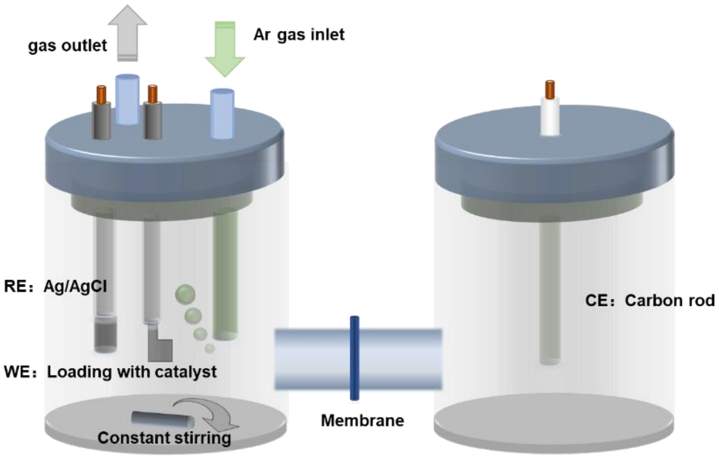

Figure S13. Illustration of H-type cell used in this study, related to Figure 2.

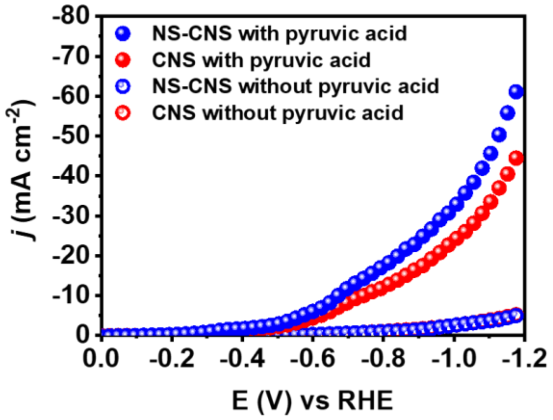

Figure S14. LSV curves of NS-CNS and CNS in different electrolyte, related to Figure 2.

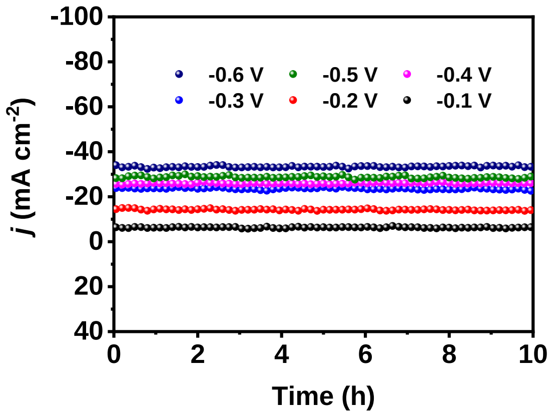

Figure S15. Chronoamperometry curves of reductive amination on NS-CNS under different potential, related to Figure 2.

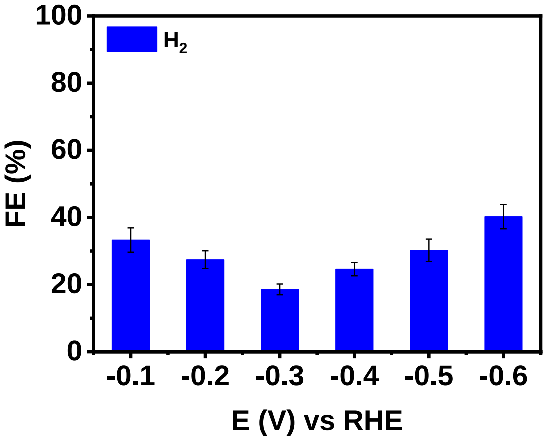

Figure S16. FE of H_2 byproducts of NS-CNS under different potential, related to Figure 2.

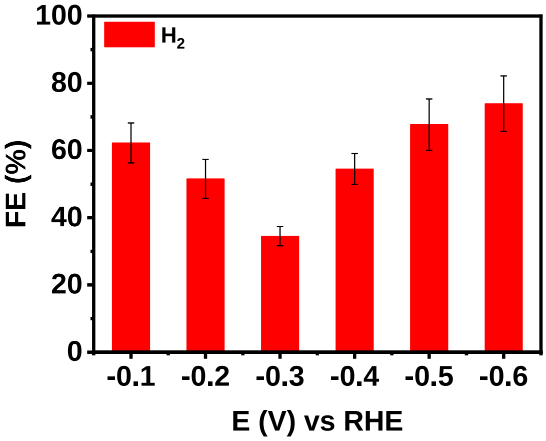

Figure S17. FE of H_2 byproducts of CNS under different potential, related to Figure 2.

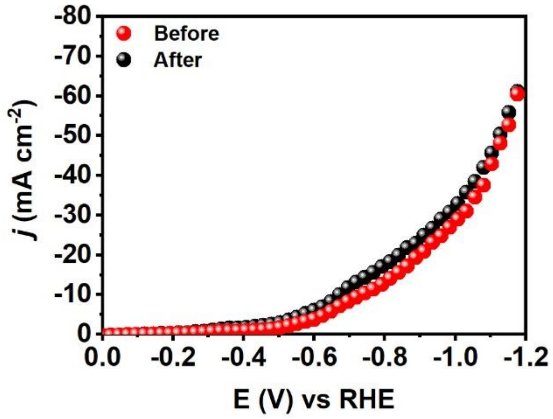

Figure S18. LSV curves of NS-CNS before and after electrolysis, related to Figure 2.

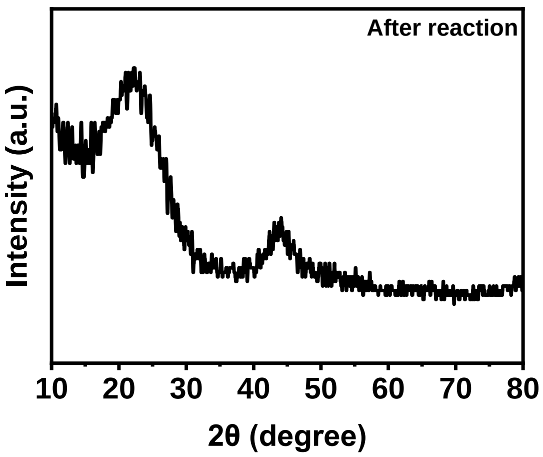

Figure S19. XRD patterns of NS-CNS after reaction, related to Figure 2.

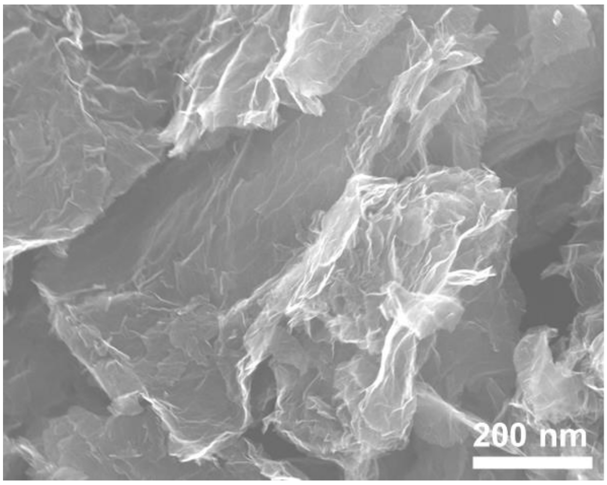

Figure S20. SEM image of NS-CNS after reaction, related to Figure 2.

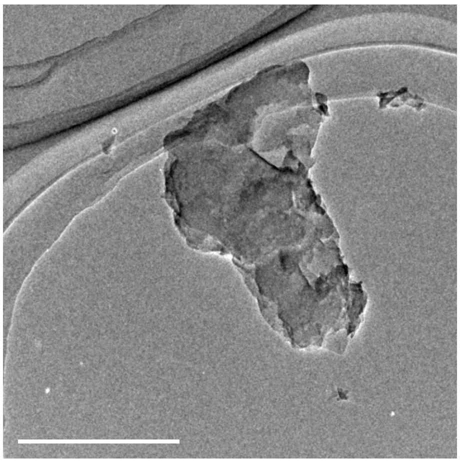

Figure S21. TEM image of NS-CNS after reaction, related to Figure 2.

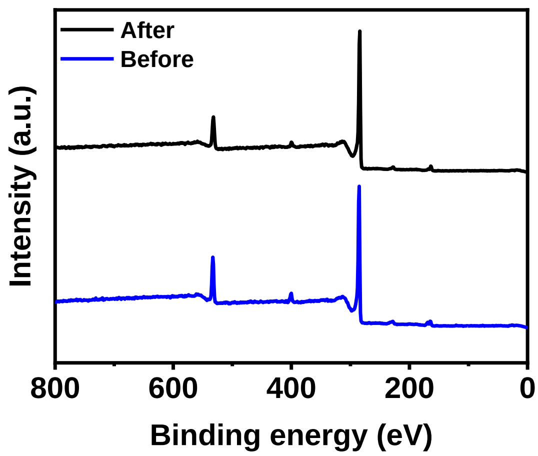

Figure S22. XPS spectra of NS-CNS after reaction, related to Figure 2.

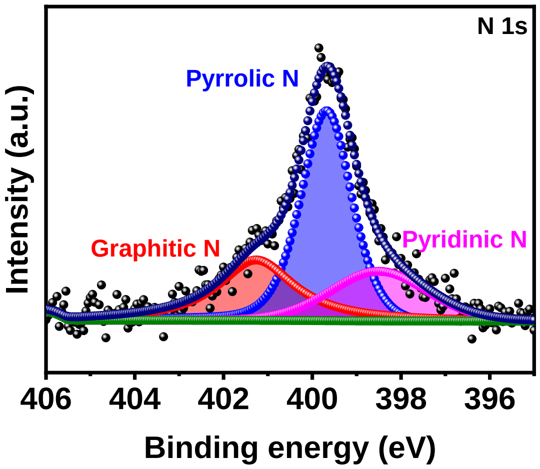

Figure S23. N 1s XPS spectra of NS-CNS after reaction, related to Figure 2.

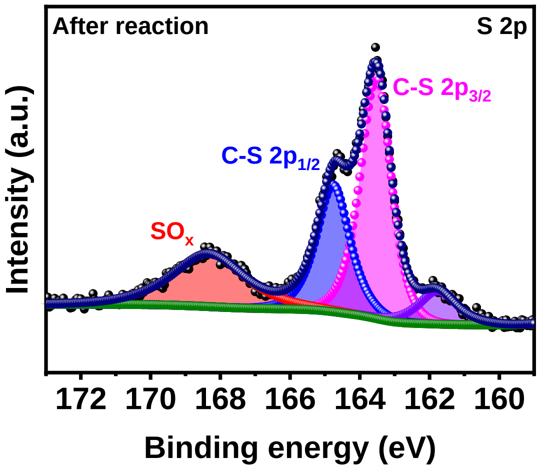

Figure S24. S 2p XPS spectra of NS-CNS after reaction, related to Figure 2.

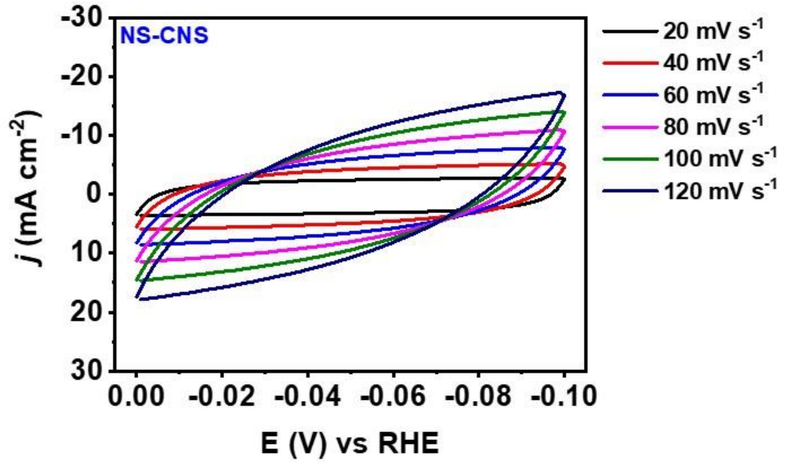

Figure S25. Cyclic voltammograms curves of NS-CNS, related to Figure 3.

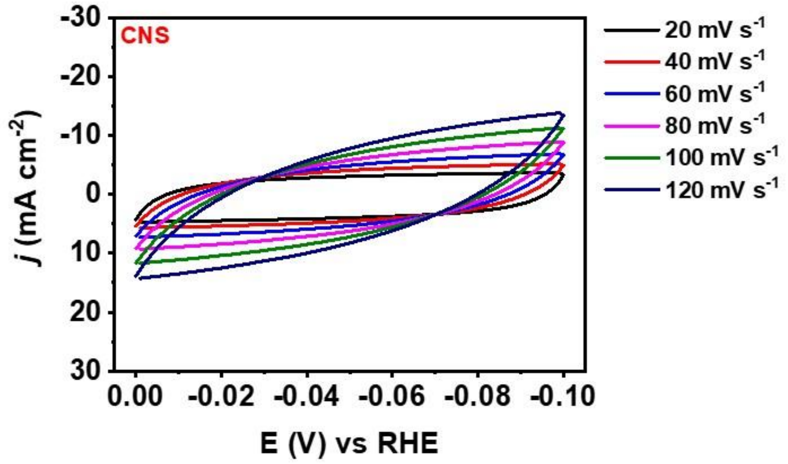

Figure S26. Cyclic voltammograms curves of CNS, related to Figure 3.

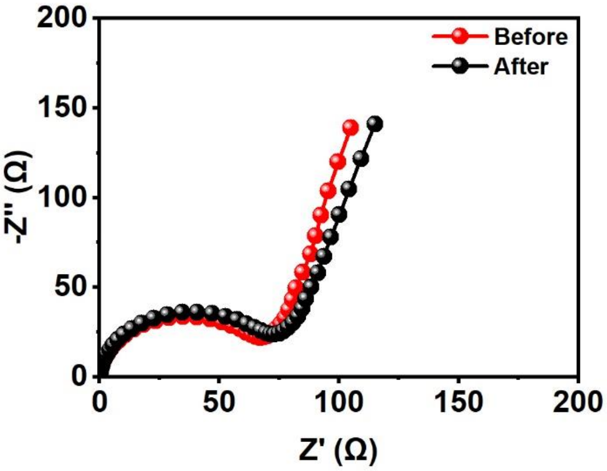

Figure S27. Nyquist plots of NS-CNS before and after electrolysis, related to Figure 3.

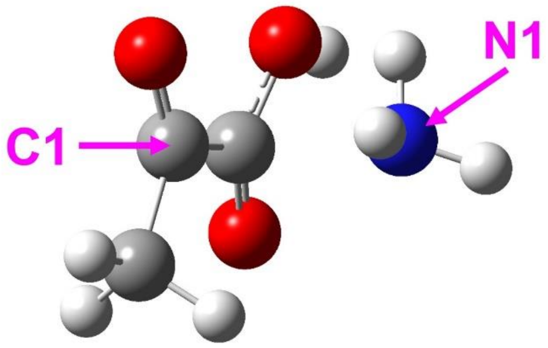

Figure S28. Illustration of transient state during formation of C-N bond, related to Figure 3.

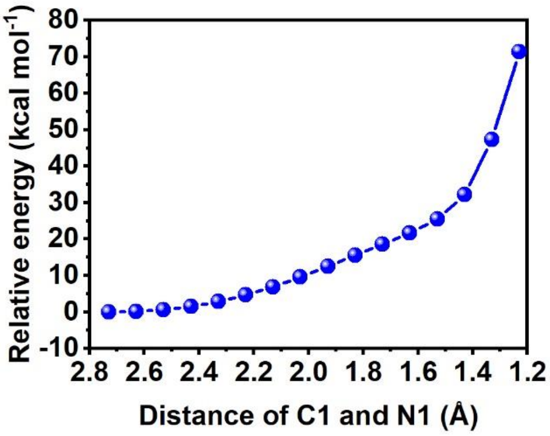

Figure S29. Relative energy of the TS structure with different distance of C and N atoms, related to Figure 3.

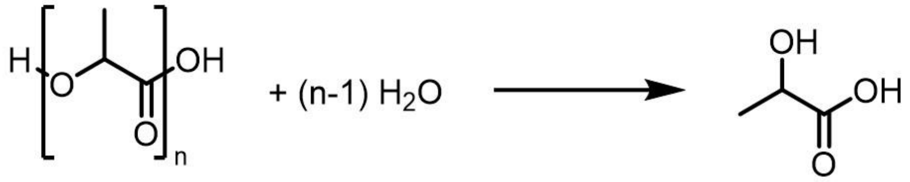

Figure S30. Hydrolysis reaction of PLA, related to Figure 4.

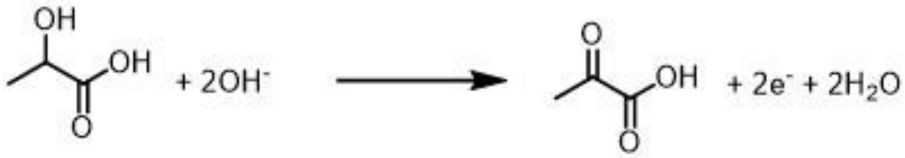

Figure S31. Electrocatalytic oxidation of lactic acid to pyruvate acid, related to Figure 4.

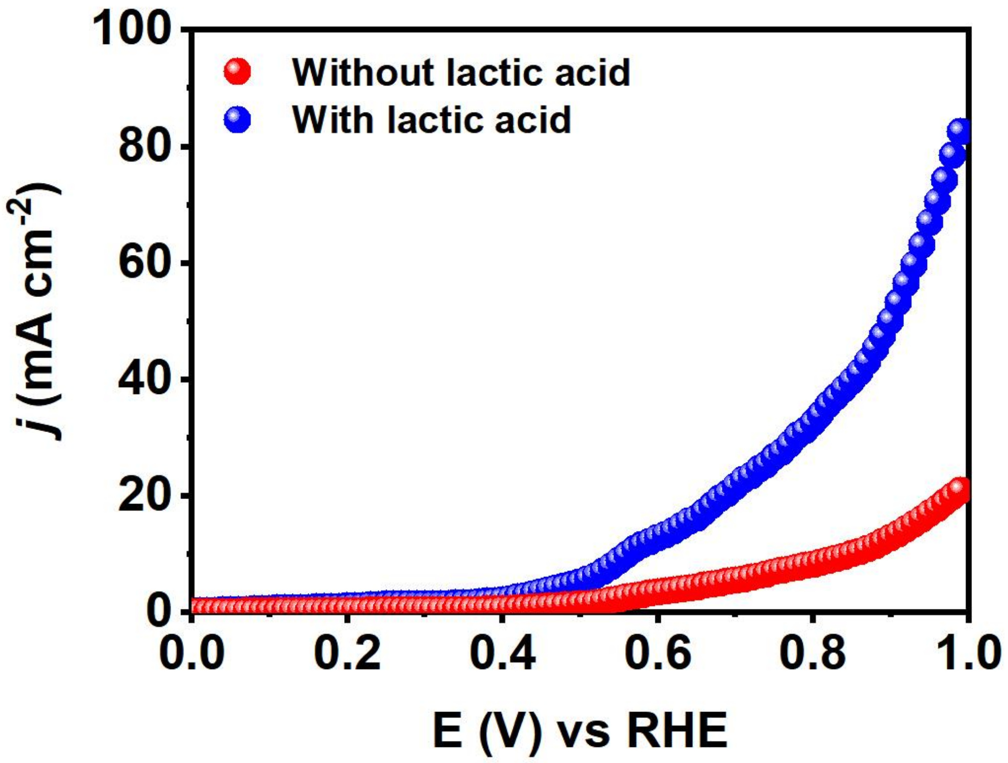

Figure S32. LSV curves of IrO_2/Ti electrode in the electrolyte with and without lactic acid substrates, related to Figure 4.

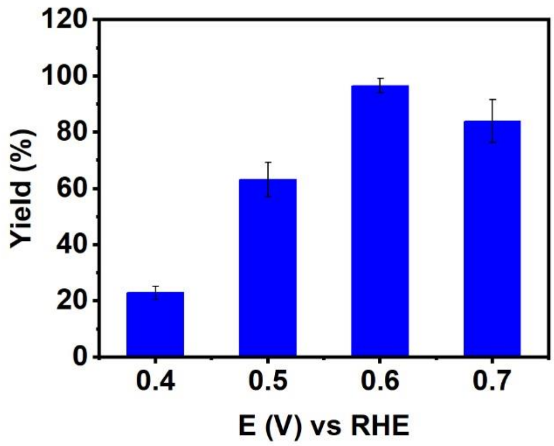

Figure S33. Production of pyruvate acid on IrO_2/Ti electrode under different potentials, related to Figure 4.
